# Malignant Rhabdoid Tumour of the Liver in the Young Adult: Report of First Two Cases

**DOI:** 10.1155/2009/628206

**Published:** 2009-09-10

**Authors:** Ettore Marzano, Emilie Lermite, Cinzia Nobili, Carlos Teyssedou, Philippe Bachellier, Jean-Pierre Arnaud, Patrick Pessaux

**Affiliations:** ^1^Centre de Chirurgie Viscérale et de Transplantation, Hôpital de Hautepierre, 67098 Strasbourg, France; ^2^Service de Chirurgie Digestive, Hôpital Universitaire, 49033 Angers, France

## Abstract

Few cases of malignant rhabdoid tumour (MRT) of the liver are reported in literature and always in paediatric patients. We report the first two cases of young adults submitted to hepatic resection for MRT of the liver. A major liver resection was performed in both cases. The histology showed round or fusiform, loosely cohesive cells. The cytoplasm contained abundant eosinophilic inclusions, which caused the nuclei to be located in eccentric locations, giving the characteristic rhabdoid appearance. The immunohistochemical study was performed, and characteristic lack of nuclear INI1 protein expression was found. In a case surgery was associated to chemoradiotherapy. One patient died at 48 months followup for tumour recurrence. The other is still alive at 25 months followup. MRTs are rare tumours of pediatric age with poor prognosis. Hypothetical less malignant behaviour in the young adults could be supposed. Therefore an aggressive surgical and oncological treatment seems justified.

## 1. Introduction

Malignant rhabdoid tumours (MRTs) are highly aggressive tumours which usually presented in the central nervous system, in the kidneys and soft tissues of children. They were firstly described in 1978 as an aggressive variant of Wilms' tumour [1]. MRT has now been accepted as a distinctive clinicopathological entity [2]. Since then identical tumours have been described in a variety of extra-renal organs. The first evidence in literature of a liver tumour with rhabdoid features was in 1982 by Gonzalez-Crussi et al. [3]. Until now few cases are reported in literature [4] and always in paediatric patients. 

We report the first two cases of young adults submitted to hepatic resection for MRT of the liver. The histological and immunohistochemical findings, the surgical treatment, and the clinical outcome are described. 

## 2. Case Report

**Case 1**

**Figure 1:**
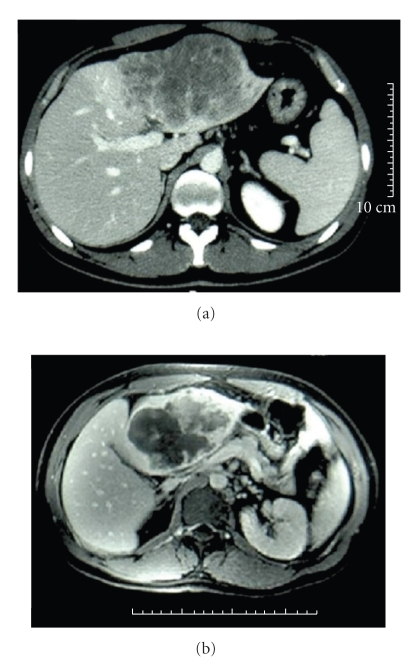
CT-scan (a) and the Magnetic Resonance Imaging (b) showed a voluminous heterogeneous tumour occupying the left liver. The injection of contrast showed a late vascularisation.

A 27-year-old male, in a good overall health, was admitted at the Emergency Unit for acute epigastric pain since 48 hours. The clinical examination of the abdomen found a mass at the palpation of the epigastric region. There was no weight loss, jaundice, diarrhoea, or vomiting. Trans-abdominal ultrasound revealed a large heterogeneous mass with intratumoral arterial vascularization, which occupied the left liver (15 × 7 cm) and the presence of peritoneal liquid in the Douglas space. The injection of contrast agent (Sonovue) showed a precocious hypervascularisation of the lesion whit hypoechogenic spots and late wash-out. No alterations of biological examinations and tumoral markers were revealed: alpha fetoprotein = 1.3 ng/mL (Normal <5), CA 19 − 9 = 15.2 U/mL (Normal <37). The CT-scan and the Magnetic Resonance Imaging (MRI) confirmed the ultrasound report and moreover showed a nodule of 1.5 cm on the segment VIII (Figures 1(a)  and 1(b)). The signal and the vascular kinetic were in favour of a fibrolamellar hepatocarcinoma or of a cholangiocarcinome. The preoperative biopsies were inconclusive. A left hepatectomy (segments II-III-IV) with atypical resection of the lesion on the segment VIII was performed, preserving middle and right hepatic vein. No postoperative complication was observed. The patient was discharged at day 7. A simple surveillance was decided by the multidisciplinary Cancer Board. A followup consisting in regular clinical consultations and CT-scan was performed. No sign of recurrence was remarked at 25-month follow-up.

**Case 2**

A 15-year-old male, in a good overall health, presented acute epigastric pain and an upper abdominal palpable mass of recent appearances. The ultrasound and the CT-scan revealed a voluminous heterogeneous tumour of 15 cm in the left liver which compressed the surrounding structures (pancreas-stomach). Considering the high risk of hemorrhagic rupture, a surgical resection was decided. The surgical exploration showed a huge capsulated hepatic tumour compressing the portal vein without signs of infiltration of vascular structure. The tumour capsule presented signs of spontaneous rupture without active bleeding. A left hepatectomy (segments II-III-IV) was performed. There were no postoperative complication, and the patient was discharged at postoperative day 10. Because of capsule rupture and risk of tumour seeding an adjuvant chemotherapy consisting in 6 cycles of IVA (ifosfamide, vincristine, actinomycin D) was proposed. At 30 months followup, the CT-scan showed two recurrence nodules involving the hepatic ligament and the peripancreatic region, measuring 6 and 7 cm, respectively. No others metastatic lesions were found. Chemotherapy consisting in 3 cycles of IVA followed by 3 cycles of adriamicine-cisplatine allowed an objective response, with mild reduction of the lesions to 4 and 5 cm, respectively. Complementary resection of the hepatic segment I and pancreaticoduodenectomy (PD) with extended lymphadenectomy was performed. The histology confirmed the recurrence of a rhabdoid tumour without lymphatic involvement. The patient underwent postoperative radiotherapy (40 Gy/20 fractions). At 43-month followup a new recurrence involving the remnant liver and the posterior bladder space was detected. A new chemotherapy with etoposide (3 cycles) was applied, followed by surgical exploration and resection of a voluminous retroperitoneal recurrence associated with right colon resection. The patient was discharged at postoperative day 15, but he died 1 month later due to a progressive cardiac failure.

### 2.1. Pathological Findings

**Figure 2:**
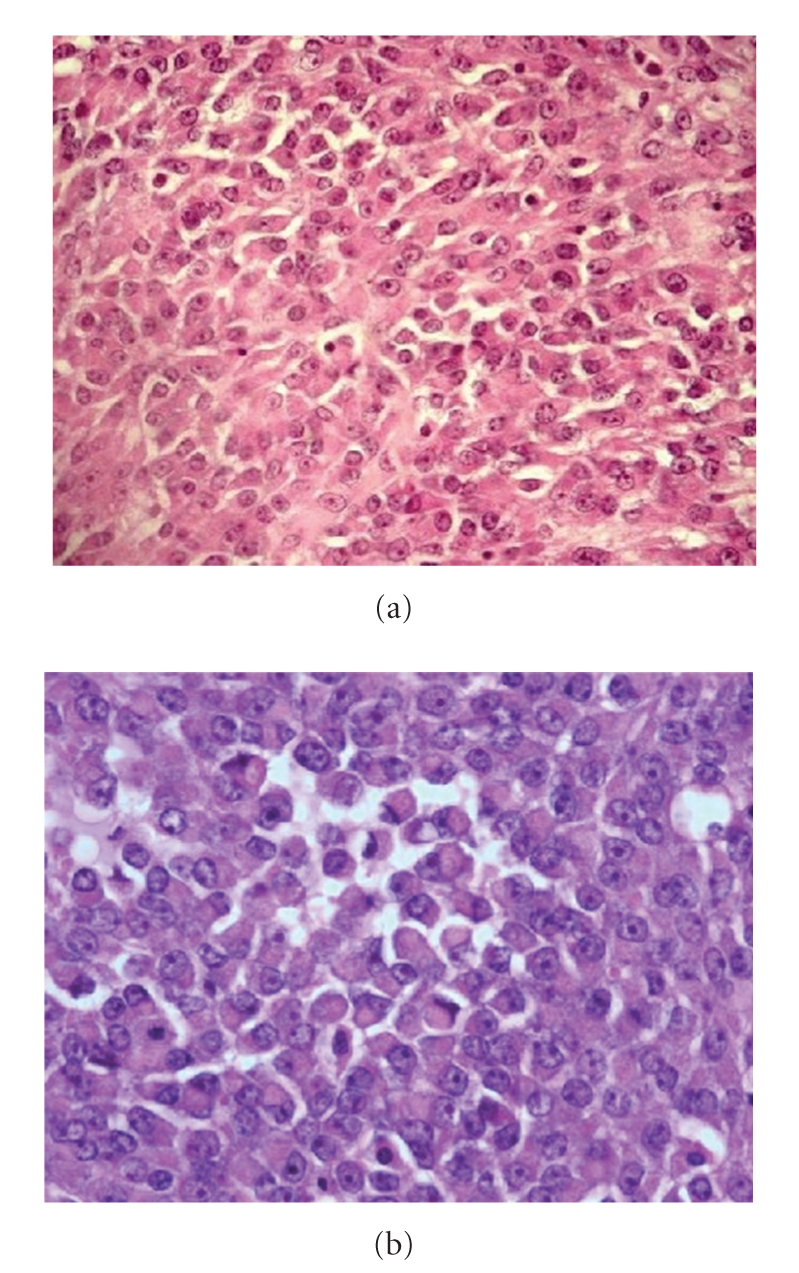
The microscopic analysis of the specimen (hemotoxylin, Phloxine, and Safran coloration) with 200 × magnification (a) and 400 × magnification (b).

The surgical specimen measured, respectively, 17 × 15 × 8 cm/weigh 942 g (Case 1) and 15 × 14 × 14 cm/weigh 1400 g (Case 2). The macroscopic examination revealed a solid whitish tumour, with necrotic and hemorrhagic areas. The microscopic study showed a lobulated uniform aspect of fibrotic septa and glandular formations. The neoplastic cells were round or fusiform, loosely cohesive, and were seen within a fibromyxoid stroma. Ovular nucleus and prominent nucleolus were observed. The number of mitosis was moderate (maximum 8 at Gx400/10 fields). The cytoplasm contained abundant eosinophilic inclusions, which caused the nuclei to be located in eccentric locations, giving the characteristic rhabdoid appearance (Figures 2(a)  and 2(b)). The cytoplasm stained positive with PAS (periodic acid-Schiff) and was digested after diastase. Necrotic and hemorrhagic foci were included within the neoplastic mass.

The immunohistochemical study was performed. The antivimentin antibody labelled the cytoplasm of almost neoplastic cells, including the cells with rhabdoid features. The anti-EMA (Epithelial Membrane Antigen) antibody diffusely marked the cellular cytoplasm with reinforcement of tumoral cells membranes. C-kit antibody significantly marked the cytoplasm of most neoplastic cells. Conversely the anti-PS-100, CK 22, CK 7, CK 19, CK 20, HMB45, desmin, myogenin, caldesmon, CD34, BCL2, synaptophysin, monoclonal NSE, and P53 antibodies did not remarkable labelled the neoplastic cells.

The researches of specific genetic translocations for Ewing tumour, alveolar rhabdomyosarcoma, synovial-sarcoma, and myxoid chondrosarcoma were negatives. Finally the immunohistochemistry demonstrated lack of nuclear INI1 protein expression. 

## 3. Discussion

 MRT was first identified in the kidney of infants and children and was described in 1978 as rhabdomyosarcomatoid variant of Wilms' tumor [1, 5]. However, because of the lack of ultrastructural or immunohistochemical evidence of myogenic differentiation, the term rhabdoid was later adapted for these neoplasms [6–8]. 

With the same morphologic and immunohistochemical features, identical tumors have been reported in a wide variety of sites including the nervous system, eye, tongue, nasopharynx, neck, mediastinum, thymus, heart, uterus, urinary bladder, vulva, skin, soft tissue, paravertebral region, and gastrointestinal tract [5, 9]. Extrarenal rhabdoid tumours, although histologically, clinically, and ultrastructurally resembling renal rhabdoid tumours, are less consistent in presentation. For this particular reason, the existence of extrarenal rhabdoid tumours of childhood as a true clinicopathologic entity has been questioned. The current consensus is that it is indeed a true but heterogenous entity. As for extrarenal rhabdoid tumours in adults, some of these tumours are identical to those seen in children, whereas others have focal areas of rhabdoid differentiation within an otherwise distinctly differentiated tumour such as a carcinoma, sarcoma, or melanoma [5]. Rhabdoid tumours characteristically are highly cellular neoplasms that show somewhat monotonous noncohesive to poorly cohesive sheets of large polyglonal cells with abundant eosinophilic cytoplasm that may contain intracytoplasmic inclusions. The nuclei are eccentric, hyperchromatic, vesicular and pleomorphic and have prominent nucleoli. Ultrastructurally, the most distinctive feature is the presence of cytoplasmic filaments. These may be of variable diameters and are arranged in bundles or whorls. The globular inclusions seen by light microscopy are composed of parallel filaments between 6 and 9 nm in diameter, packed in concentric whorls that often have lipid droplets or mitochondria at the center [5, 9]. Imunohistochemically, the rhabdoid cells express both cytokeratin and vimentin and lack myogenic differentiation [5, 6, 10]. Recent studies show that rhabdoid differentiation represents a clonal evolution in tumours of differing histogenesis. Cytogenic studies have shown that classic rhabdoid tumours contain a characteristic chromosomal abnormality involving chromosome 22, leading to mutation of Hsnf5/INII gene, located on chromosomal band 22q11.2, with lack of expression on INI1 protein at immunohistochemistry [6, 10–14]. Such a way a genetic diagnosis is possible [15].

In summary, primary malignant rhabdoid tumours of various organs have been described in literature. Only rare cases are seen in the gastrointestinal tract, in particular, the liver. These poorly differentiated neoplasms may pose a great deal of difficulty in the diagnosis because there is no glandular, squamous, or any other differentiation. In addition, it is almost impossible to make the distinction whether the tumour is of primary origin or represents a metastasis from rhabdoid tumour of a different site. In the two cases no other extrahepatic lesions were found at pre-operative imaging studies and surgical exploration. 

Liver rhabdoid tumour is a rare histological finding especially in young adults. In a review article published in 2004, Yuri et al. [4] found 19 cases with a median age of diagnosis of 16,7 months. Moreover nearly all cases (89%) occurred in the first 2 years of life, with no gender difference. Although one report showed a 6-year survival [16], the outcome of the other published cases was uniformly fatal, despite aggressive treatment. The overall mortality rate was 89% with a mean survival of 15.3 weeks. Furthermore Tekautz et al. [17] analysed a population of 31 patients affected by teratoid/rhabdoid tumours of the central nervous system (CNS). They noticed a better response to chemotherapy, a better two-years event-free survival (EFS) and overall-free survival (OS) for patient 3 years or older, compared to patients younger than 3 years (EFS 78% ± 14% versus 11% ± 6%) (OS 89% ± 11 versus 17% ± 8). In our study we reported a followup of 25 months without signs of recurrence for Case 1. Case 2 had a disease free survival of 31 months and an overall survival of 48 months. Probably a less aggressive behaviour of MRT in later presentation compared to infant could be supposed.

As concern chemoradiotherapy, no consistently effective regimen has yet been established. Ifosfamide, vincristine, cyclophosfamide, etoposide, epirubicin and actinomycin D based chemotherapies are the treatments of choice. Banzai et al. [18] reported an effective response to combination of ifosfamide-epirubicin-cisplatine for MRT of the ovary. Tekautz et al. [17] described a regimen of high-dose alkylator-based chemotherapy associated to radiotherapy, followed by autologous bone marrow transplantation for MRT of CNS. Satisfactory results were achieved in patients 3 years and older. However a common consensus was not reached, and a standard regimen is not yet established. In Case 2, an association of surgery and chemoradiotherapy was performed. This aggressive attitude permitted an acceptable control of the recurrence disease with 48-month survival. 

The aggressive and incurable nature of rhabdoid tumors corroborates the need to develop a targeted and effective therapy for these tumors. Rhabdoid tumors and cells are exquisitely dependent on cyclin D1 for genesis and survival, suggesting that targeting the cyclin/cyclin-dependent kinase (cdk) axis may be an effective therapeutic strategy for these tumors. Currently, there are several studies regarding the use of a pan-cdk inhibitor, flavopiridol, that both inhibits cdk activity and transcriptionally down-modulates cyclin D1 which may effectively inhibit rhabdoid tumor growth. Flavopiridol is potentially a novel chemiotherapeutic agent for rhabdoid tumors [19].

In conclusion MRTs are rare liver tumours. Histological diagnosis is not always easily achieved, but lack of expression on INI1 protein at immunohistochemistry could be helpful. Usually liver rhabdoid tumours developed in pediatric patients and in most of the cases the prognosis is very poor. Presentation in young adults is exceptional, and they probably have a better biological behaviour. As concern chemoradiotherapy no consistently effective regimen has yet been established. Aggressive surgical conduct seems justified also in case of recurrence disease.
